# Supplementary material for: Polycomb repressive complex 2 shields naïve human pluripotent cells from trophectoderm differentiation
Source: Nat Cell Biol. 2022 May 30;24(6):845–57. doi: 10.1038/s41556-022-00916-w (PMC9203276; doi:10.1038/s41556-022-00916-w)
Supplement: Supplementary file 1 — Reporting Summary [file 41556_2022_916_MOESM1_ESM.pdf]

nature portfolio

Corresponding author(s): Simon Elsasser
Fredrik Lanner

Last updated by author(s): Feb 4, 2022

Reporting Summary

Nature Portfolio wishes to improve the reproducibility of the work that we publish. This form provides structure for consistency and transparency in reporting. For further information on Nature Portfolio policies, see our Editorial Policies and the Editorial Policy Checklist.

Statistics

For all statistical analyses, confirm that the following items are present in the figure legend, table legend, main text, or Methods section.

n/a	Confirmed	
☐	☒	The exact sample size (*n*) for each experimental group/condition, given as a discrete number and unit of measurement
☐	☒	A statement on whether measurements were taken from distinct samples or whether the same sample was measured repeatedly
☐	☒	The statistical test(s) used AND whether they are one- or two-sided<br>*Only common tests should be described solely by name; describe more complex techniques in the Methods section.*
☒	☐	A description of all covariates tested
☐	☒	A description of any assumptions or corrections, such as tests of normality and adjustment for multiple comparisons
☐	☒	A full description of the statistical parameters including central tendency (e.g. means) or other basic estimates (e.g. regression coefficient) AND variation (e.g. standard deviation) or associated estimates of uncertainty (e.g. confidence intervals)
☐	☒	For null hypothesis testing, the test statistic (e.g. *F*, *t*, *r*) with confidence intervals, effect sizes, degrees of freedom and *P* value noted<br>*Give P values as exact values whenever suitable.*
☒	☐	For Bayesian analysis, information on the choice of priors and Markov chain Monte Carlo settings
☒	☐	For hierarchical and complex designs, identification of the appropriate level for tests and full reporting of outcomes
☐	☒	Estimates of effect sizes (e.g. Cohen's *d*, Pearson's *r*), indicating how they were calculated

Our web collection on statistics for biologists contains articles on many of the points above.

Software and code

Policy information about availability of computer code

Data collection	For ChIP-seq data collection, Illumina NextSeq500 platform with paired-end settings was used. FASTQ files were obtained from Illumina BaseSpace.RNA-seq data collection through bgi service https://www.bgi.com/global/index.scRNA-seq: cDNA libraries were sequenced using Illumina Nextseq 2000 Platform 100 cycles P2 and Illumina Nextseq 550 High Output kit v2.5 (150 Cycles). More information can be found in the methods section of the manuscript.
Data analysis	ChIP-seq data primary analysis was done using minute, a workflow for multiplexed ChIP analysis: https://github.com/NBISweden/minute.RNA-seq data primary analysis was done using standard rnaseq analysis pipeline (v2.0) from nf-core: https://nf-co.re/rnaseq . Differential expression analysis was done using DESeq2. scRNA-seq raw reads were aligned to the human GRCh38 reference genome (v.3.0.0, GRCh38, from the 10X Genomics website) using Cell Ranger v6.1.1 with default settings for the 'cellranger multi' pipeline (10X Genomics).Details in the workflow steps on the methods section of the manuscript.<br>Downstream analysis performed with a broad set of open-source publicly available R v4.1.2 libraries: ggplot2 v3.3.5, ggpubr v0.4.0, DESeq2 v1.34.0, tidyverse v1.3.1, rtracklayer v1.54.0, workflowr v1.6.2, wigglescout v0.13.5, seurat v3.1.4, scran v1.14.6, batchelor v1.2.4.<br>Supplementary code for the downstream analysis and figure generation is available at https://github.com/elsasserlab/hesc-epigenomics.<br>scRNA-seq data can be browsed at https://petropoulos-lanner-labs.clintec.ki.se/app/shinyEZH2i. Trajectory analysis was performed using monocle2 v2.14.0.

For manuscripts utilizing custom algorithms or software that are central to the research but not yet described in published literature, software must be made available to editors and reviewers. We strongly encourage code deposition in a community repository (e.g. GitHub). See the Nature Portfolio guidelines for submitting code & software for further information.

Data

Policy information about availability of data

All manuscripts must include a data availability statement. This statement should provide the following information, where applicable:

- Accession codes, unique identifiers, or web links for publicly available datasets
- A description of any restrictions on data availability
- For clinical datasets or third party data, please ensure that the statement adheres to our policy

The high-throughput data reported in this study have been deposited in GEO under the accession number GSE181244, which includes demultiplexed and deduplicated reads and a quantitatively scaled bigwig track for each sample. Previously published datasets that were re-analysed here, including scRNA-seq data from 3 human embryonic datasets and one dataset of post-implantation amniotic sac embryoid (PASE) (E-MTAB-3929 86, GSE136447 68, E-MTAB-9388 70, and GSE134571 69) are listed in Supplementary Table 1.

Field-specific reporting

Please select the one below that is the best fit for your research. If you are not sure, read the appropriate sections before making your selection.

☒ Life sciences ☐ Behavioural & social sciences ☐ Ecological, evolutionary & environmental sciences

For a reference copy of the document with all sections, see nature.com/documents/nr-reporting-summary-flat.pdf

Life sciences study design

All studies must disclose on these points even when the disclosure is negative.

Sample size	No statistical test was performed to determine sample size. Sample size for omics experiments (3 biological replicates for ChIP seq, bulk and sc RNA seq) was determined based on typical ranges used in the field (https://genome.cshlp.org/content/genome/22/9/1813.full.html.)
Data exclusions	No data were excluded for the analyses.
Replication	The ChIP-seq, Bulk and sc-RNA seq were performed once with 3 biological replicates within each of the experiments. All other experiments in the study were repeated in separate batches and are reported in the respective figure legends.
Randomization	For cell culture experiments, treatment groups were attributed randomly between wells. Within the MINUTE-ChIP and sc RNA seq experiment barcodes were assigned randomly between samples. IF images were also acquired with no particular bias. For other experiments in the study randomization was not applicable due to the nature of the protocol.
Blinding	No blinding was done

Reporting for specific materials, systems and methods

We require information from authors about some types of materials, experimental systems and methods used in many studies. Here, indicate whether each material, system or method listed is relevant to your study. If you are not sure if a list item applies to your research, read the appropriate section before selecting a response.

Materials & experimental systems

n/a	Involved in the study	
☐	☒	Antibodies
☐	☒	Eukaryotic cell lines
☒	☐	Palaeontology and archaeology
☒	☐	Animals and other organisms
☒	☐	Human research participants
☒	☐	Clinical data
☒	☐	Dual use research of concern

Methods

n/a	Involved in the study	
☐	☒	ChIP-seq
☒	☐	Flow cytometry
☒	☐	MRI-based neuroimaging

Antibodies

Antibodies used

Primary antibodies :
Immunoflourescence
GATA3 clone L50-823 (1:200, BD; 558686)
H3K27me3 C36B11 (1:500, Cell Signaling Technologies; 9733S)
OCT4 (1:200, SantaCruz; sc-5279)
SOX2 clone EP103 (1:3, Biogenex; AN833)

	NANOG (1:200, RnD; AF1997-SP)<br>EED (E4L6E) XP® (1:200, Cell Signaling Technology; 85322)<br>Immunoblotting<br>H3 (1:10,000,Active motif 39763)<br>H3K4me3 (1: 5000, Millipore 04-745)<br>H3K27me3 (1: 5000, Millipore 07-449)<br>H2Aub (1:5000, Cell Signaling 8240S)<br><br>Secondary antibodies:<br>Immunofluorescence<br>donkey a-mouse IgG (H+L) Alexa fluor 555, donkey a-rabbit IgG (H+L) Alexa fluor 647, donkey a-goat IgG (H+L) Alexa fluor 647, donkey a-mouse IgG (H+L) Alexa fluor 488, donkey a-rabbit IgG (H+L) Alexa fluor 555<br>(all from Thermofisher; A-31570, A-31573, A-21447, A-21202 and A-31572, respectively)<br>Immunoblotting<br>IRDye® 680RD anti-rabbit and IRDye® 800CW anti-mouse (LI-COR) at 1:5000 dilution
Validation	All antibodies were previously validated by vendors and/or published work.<br>GATA3 clone L50-823 (BD; 558686): https://www.bdbiosciences.com/en-au/products/reagents/microscopy-imaging-reagents/immunofluorescence-reagents/purified-mouse-anti-gata3.558686; cited in 7 publications<br>H3K27me3 C36B11 (Cell Signaling Technologies; 9733S): https://www.cellsignal.com/products/primary-antibodies/tri-methyl-histone-h3-lys27-c36b11-rabbit-mab/9733; cited in 759 publications<br>OCT4 (SantaCruz; sc-5279): https://www.scbt.com/sv/p/oct-3-4-antibody-c-10; cited in 2201 publications<br>SOX2 clone EP103 (Biogenex; AN833): https://biogenex.com/wp-content/uploads/2019/11/932-833N.pdf; cited in 4 publications<br>NANOG (RnD; AF1997-SP): https://www.rndsystems.com/products/human-nanog-antibody_af1997; cited in 166 publications<br>EED (E4L6E) XP® (Cell Signaling Technology; 85322): https://www.cellsignal.com/products/primary-antibodies/eed-e4l6e-xp-rabbit-mab/85322; cited in 4 publications<br>H3 (Active motif 39763): https://www.activemotif.com/catalog/details/39763; cited in 23 publications<br>H3K4me3 (Millipore 04-745) : https://www.merckmillipore.com/SE/en/product/Anti-trimethyl-Histone-H3-Lys4-Antibody-clone-MC315-rabbit-monoclonal,MM_NF-04-745; cited in 98 publications<br>H3K27me3 (Millipore 07-449): https://www.merckmillipore.com/SE/en/product/Anti-trimethyl-Histone-H3-Lys27-Antibody,MM_NF-07-449?ReferrerURL=https%3A%2F%2Fwww.google.com%2F; cited in > 200 publications<br>H2Aub (Cell Signaling 8240S): https://www.cellsignal.com/products/primary-antibodies/ubiquityl-histone-h2a-lys119-d27c4-xp-rabbit-mab/8240; cited in 230 publications.

Eukaryotic cell lines

Policy information about cell lines

Cell line source(s)	H9 (Wicell; WA09), HS975 (inhouse; DOI: 10.1038/ncomms4195, PMID: 24463987) , mouse inactivated embryonic fibroblasts (Gibco; A24903)
Authentication	Cell lines were not further authenticated.
Mycoplasma contamination	All the cell lines are tested negative for mycoplasma contamination.
Commonly misidentified lines<br>(See ICLAC register)	No cell line used in this paper is listed in the ICLAC database.

ChIP-seq

Data deposition

☒ Confirm that both raw and final processed data have been deposited in a public database such as GEO.

☒ Confirm that you have deposited or provided access to graph files (e.g. BED files) for the called peaks.

Data access links<br>*May remain private before publication.*	GSE181244: https://www.ncbi.nlm.nih.gov/geo/query/acc.cgi?acc=GSE181244
Files in database submission	MINUTE-ChIP: Demultiplexed FASTQ files, scaled bigWig files.<br>RNA-seq: FASTQ files, normalized bigWig files.
Genome browser session<br>(e.g. UCSC)	n/a

Methodology

Replicates	Three biological replicates
Sequencing depth	ChIP seq replicates were sequenced at a average depth of 6 million paired-end reads.
Antibodies	5 ug each of H3K27me3 {Millipore 07-449}, H3K4me3 {Millipore 04-745} and H2AUb {Cell Signaling 8240S}

Peak calling parameters	No peak calling was done for this analysis.
Data quality	Multiple QC checks were performed throughout the analysis (FastQC, Picard repeat stats, insert size, estimated library size).
Software	All software used is listed in the methods section of the manuscript. minute pipeline is paired with a conda environment for reproducibility. nf-core RNA-seq primary analysis pipeline was pulled from the repository and run on a Singularity container. Downstream analysis is available on GitHub and rendered as a website with workflowr v1.6.2.
