# Supplementary material for: Polycomb repressive complex 2 shields naïve human pluripotent cells from trophectoderm differentiation
Source: Nat Cell Biol. 2022 May 30;24(6):845–57. doi: 10.1038/s41556-022-00916-w (PMC9203276; doi:10.1038/s41556-022-00916-w)

Original blots of representative gel image in Extended Data Fig. 2c

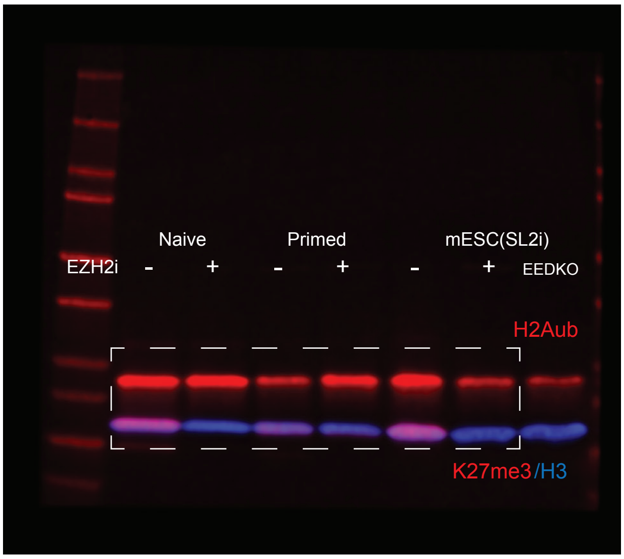

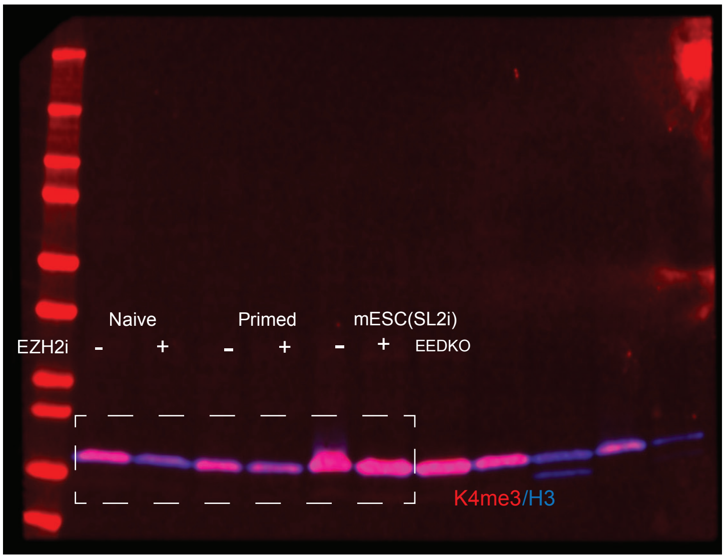

Original blots of duplicates used to plot chart in Extended Data Fig. 2c

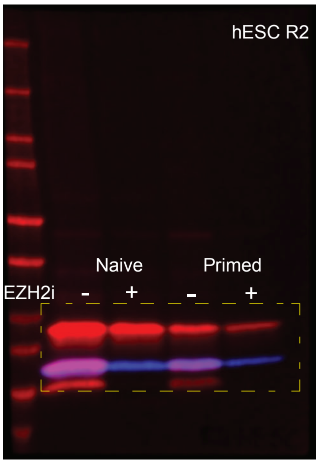

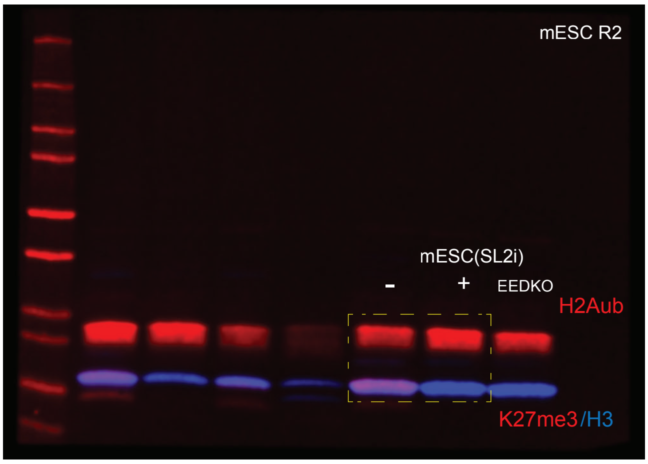

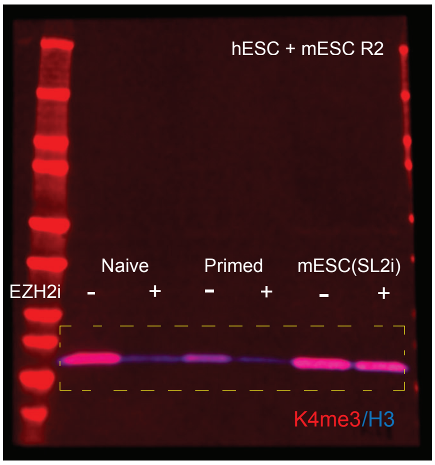

Supplement: Supplementary file 12 — Uncropped blots corresponding to Extended Data Fig. 2. [file 41556_2022_916_MOESM12_ESM.pdf]
